# Case Report: Immune checkpoint inhibitor-associated myocarditis in an esophageal cancer patient with myasthenia gravis following combined radiotherapy and immunotherapy

**DOI:** 10.3389/fonc.2025.1652084

**Published:** 2026-01-02

**Authors:** Xue Ren, Defu Yang, Ying Xu, Ying Yan

**Affiliations:** Department of Radiation Oncology, General Hospital of Northern Theater Command, Shenyang, China

**Keywords:** case report, esophageal cancer, immune checkpoint inhibitors (ICIs), immunotherapy, radiotherapy

## Abstract

Myocarditis associated with immune checkpoint inhibitors is a rare but potentially fatal immune-related adverse event. Esophageal cancer patients with myasthenia gravis, who are recommended to receive radiotherapy combined with immunotherapy, are at risk for ICI-related myocarditis. We report a 69-year-old male esophageal cancer patient with myasthenia gravis who was diagnosed with immune checkpoint inhibitor related myocarditis after receiving radiotherapy combined with immunotherapy. The patient’s laboratory test results showed elevated troponin and N-terminal pro-B-type natriuretic peptide. Electrocardiography revealed arrhythmia and complete left bundle branch block. Despite treatment with methylprednisolone, the patient’s condition was severe, and clinical and auxiliary examination symptoms continued to deteriorate, leading to his unfortunate demise. In this case, the adverse event of myocarditis induced by radiotherapy combined with immunotherapy in oncology patients with myasthenia gravis is an area that requires further investigation. Clinicians must carefully weigh the potential benefits and risks when considering this combined treatment approach and closely monitor patients for adverse events.

## Introduction

Immune checkpoint inhibitors (ICIs), including anti-programmed cell death protein-1 (PD-1), anti-programmed cell death ligand-1 (PD-L1), anti-cytotoxic T-lymphocyte-associated protein-4 (CTLA-4), and lymphocyte activation gene-3 (LAG-3) antibodies, have become an integral component of treatment for more than 20 types of cancer by releasing inhibitory signals on antitumor immunity and enhancing T cell–mediated immune responses (1). These agents can induce a broad spectrum of toxicities driven by dysregulated immune activation, collectively referred to as immune-related adverse events (irAEs). Common irAEs include thyroiditis and hepatitis (2), whereas myocarditis, myositis, and overlapping myasthenia gravis (MG) represent rare but potentially fatal complications (3). Radiotherapy (RT) remains a cornerstone local modality that uses high-energy ionizing radiation to induce DNA damage in tumor cells and thereby achieve local control or eradication (4). MG is a chronic autoimmune disorder that primarily affects the neuromuscular junction, and myocarditis is among the most severe complications encountered in patients with MG. A systematic review showed that among 35 patients with coexisting MG and myocarditis, 40% of myocarditis cases were attributed to ICIs (3). The prognosis of MG complicated by myocarditis is poor; in that study, 18 of 35 patients died during hospitalization (5). For patients with MG receiving ICIs, close monitoring of cardiac enzymes and other relevant indicators is therefore crucial, and in those with persistent dyspnea or difficulty weaning from mechanical ventilation, the possibility of concomitant myocarditis should be carefully considered (6). Myocarditis is a particularly concerning cardiovascular condition, with a reported mortality of up to 50% and severe cardiovascular complications occurring in as many as 46% of patients (7). ICI-associated myositis also carries substantial risk, with a reported fatality rate of approximately 21% and nearly half of affected patients experiencing severe complications (8). Overall, approximately 1% of patients treated with ICIs develop myocarditis; among these, about 25% also develop myositis and 11% develop MG (9). The simultaneous occurrence of myocarditis, myositis, and MG is exceedingly rare and has been described mainly in case reports and small case series (1). These irAEs can involve almost any organ system, including the cardiovascular, neurological, and neuromuscular systems (10). Here, we report a case of a patient with esophageal cancer and pre-existing MG who developed ICI-related myocarditis following combined RT and immunotherapy, and we discuss the diagnostic approach, management, and clinical outcome.

## Case description

**Figure 1:**
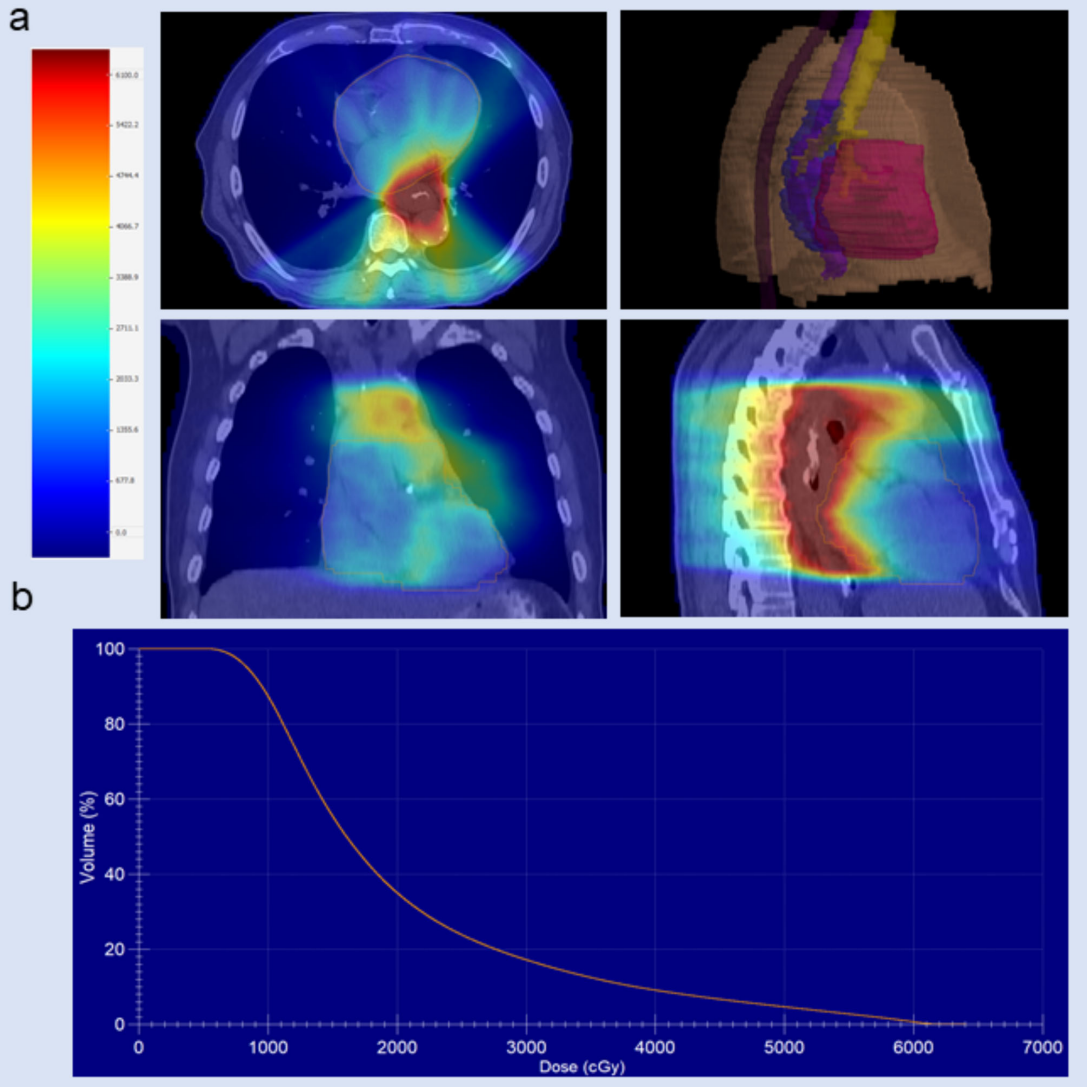
Distribution of radiation dose to the heart. **(a)** Axial, coronal, and sagittal views of the radiation dose distribution, along with a 3D plan evaluation. **(b)** Dose-volume histogram (DVH) for the heart.

**Figure 2:**
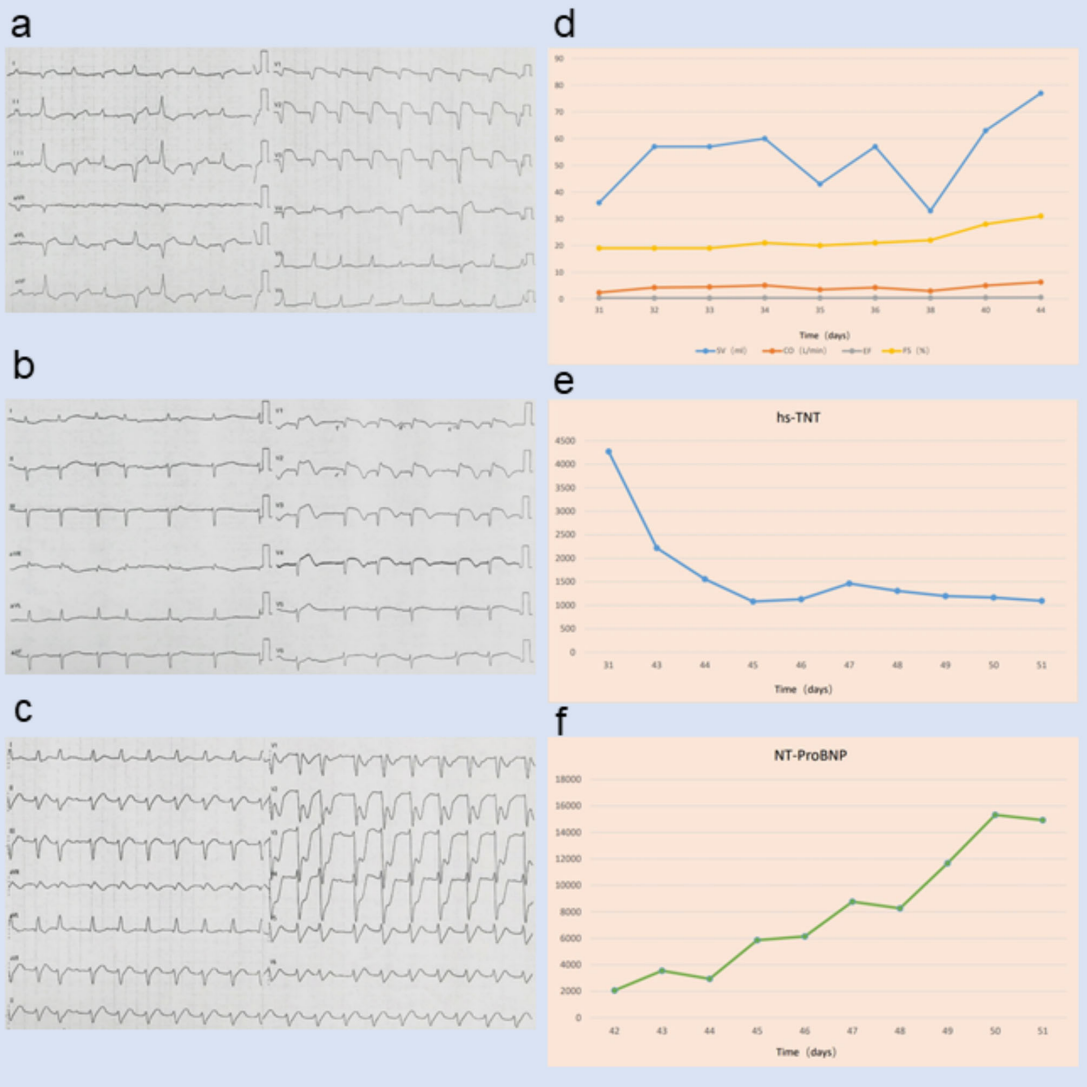
Electrocardiographic and laboratory findings. **(a)** ECG on day 1 of symptom onset (31 days post-immunotherapy) showing tachycardia and complete left bundle branch block. **(b)** ECG(40 days post-immunotherapy)showing tachycardia, premature ventricular contractions, and incomplete right bundle branch block. **(c)** ECG on the day of death (51 days post-immunotherapy) showing atrial fibrillation, right bundle branch block with left anterior fascicular block, and low QRS voltage. **(d)** Echocardiogram demonstrating changes in left ventricular systolic function following treatment. **(e)** Serial high-sensitivity troponin T (hs-TnT) levels. **(f)** Serial N-terminal pro-B-type natriuretic peptide (NT-proBNP) levels.

A 69-year-old man was diagnosed with esophageal squamous cell carcinoma. His medical history included hypertension and coronary heart disease for more than 20 years, as well as a 4-year history of generalized MG, characterized by right-sided ptosis, diplopia, and positivity for anti–acetylcholine receptor antibodies (AChR-Ab). His MG symptoms had been controlled with pyridostigmine (60 mg three times daily), and no respiratory involvement was documented prior to initiation of ICI therapy. In September 2024, the patient commenced thoracic RT with a planned total dose of 5,800 cGy. Cardiac radiation exposure was within the planned dosimetric constraints (heart volume 844 cm³; maximum dose 6,292 cGy; minimum dose 519 cGy; mean dose 2,025 cGy; V60 < 1%) (**Figure 1**). During this period, he received two cycles of Sintilimab, each at a dose of 200 mg. Baseline cardiac enzymes and electrocardiogram (ECG) findings were normal before RT. Thirty-one days after initiation of immunotherapy, and after 19 fractions of RT—before completion of the planned course, at a time when the cumulative heart dose remained below the planned total—the patient developed sudden chest tightness, shortness of breath, and palpitations. On physical examination, oxygen saturation was 96%, respiratory rate 20 breaths/min, blood pressure 140/101 mmHg, heart rate 101 beats/min, and body temperature was normal. Laboratory tests showed markedly elevated serum creatine kinase (CK 2,601 U/L; reference range 0–190), creatine kinase MB isoenzyme (CK-MB 37.3 U/L; reference 0–25), high-sensitivity troponin T (hs-TnT 4,269 ng/mL; reference <14), and N-terminal pro–B-type natriuretic peptide (NT-proBNP 2,061 pg/mL; reference <125), together with alanine aminotransferase 187 U/L (reference 0–41) and aspartate aminotransferase 455 U/L (reference 0–40). ECG demonstrated tachycardia with complete left bundle branch block (**Figure 2a**). Transthoracic echocardiography with tissue Doppler imaging (TDI) revealed new, mildly reduced left ventricular systolic function (stroke volume 60 mL, cardiac output 5.1 L/min, ejection fraction approximately 50%, fractional shortening 25%) (**Figure 2d**). Coronary angiography showed multiple coronary stenoses, with collateral flow from the right coronary artery to the left circumflex artery (RCA–LCX) via a second-degree collateral. In the context of recent ICI exposure, the pattern of biomarker elevation, and the new-onset ECG and echocardiographic abnormalities, ICI-related myocarditis with concomitant myositis was suspected. The patient was transferred to the intensive care unit for continuous cardiac monitoring (telemetry) and daily transthoracic echocardiography. He subsequently developed acute liver failure and underwent plasma exchange. Liver function tests showed partial improvement in transaminases, whereas bilirubin levels continued to rise, indicating severe hepatic injury with cholestasis. The liver enzyme abnormalities were considered to be related to systemic inflammatory injury and cardiac dysfunction. The patient also developed acute kidney injury and required dialysis. During this period, intermittent diplopia, ptosis, proximal muscle weakness, and increased muscle pain were observed, suggesting exacerbation of MG with associated myositis. After multidisciplinary consultation, the clinical presentation and ancillary findings were considered consistent with severe immune-related myocarditis with overlapping features of MG. High-dose corticosteroid therapy was initiated immediately (methylprednisolone 500 mg once daily for 5 days), after which left ventricular function improved, troponin levels decreased, and NT-proBNP levels initially rose and then gradually declined (**Figures 2e, f**). The patient experienced multiple episodes of ventricular tachycardia, which were managed with electrical cardioversion. Owing to progressive multiorgan failure, he was transferred to the department of critical care medicine. Vasoactive agents, systemic corticosteroids, anti-infective and anti-inflammatory therapies, continuous renal replacement therapy, double plasma molecular adsorption system, and other organ-supportive measures were administered. Despite one week of intensive treatment and resuscitative efforts, the patient developed sudden cardiac arrest and was pronounced dead.

## Discussion

The diagnosis of ICI-associated myocarditis, myositis, and MG relies predominantly on the integration of clinical presentation and laboratory biomarkers. Cardiac magnetic resonance imaging is the imaging modality of choice for suspected myocarditis, whereas endomyocardial biopsy remains the diagnostic gold standard (11). In the present case, the diagnosis was established mainly on the basis of the temporal relationship with ICI administration, markedly elevated cardiac biomarkers, characteristic ECG abnormalities, and echocardiographic findings, all occurring in the setting of pre-existing MG. Glucocorticoids are the mainstay of treatment for MG complicated by myocarditis and were promptly initiated in this patient (12). Nevertheless, despite timely standard therapy and comprehensive multidisciplinary support, the patient’s condition progressed to fatal multiorgan failure, underscoring the potentially fulminant nature of this overlap syndrome in high-risk individuals.

The concurrence of MG and myocarditis in this case highlights a critical and clinically relevant interaction. Although ICI-associated myocarditis is rare, pre-existing MG has been identified as a major risk factor for both its occurrence and severity. A recent study in the European Heart Journal reported that pre-existing MG was associated with a substantially increased risk of severe ICI-associated myocarditis, with a hazard ratio of 3.2 (95% CI 1.8–5.7) (13). The underlying pathophysiological link may involve shared autoimmune mechanisms. Both MG and myocarditis may be driven by cross-reactive T-cell responses directed against antigens expressed in skeletal and cardiac muscle, such as troponin. ICIs may disrupt peripheral immune tolerance, unleash autoreactive T-cell clones, and thereby trigger simultaneous inflammatory injury at the neuromuscular junction and within the myocardium (3, 9). This case exemplifies this particular vulnerability and emphasizes the importance of intensive cardiac surveillance in patients with MG receiving ICI-based regimens.

In this patient’s treatment course, the documented mean cardiac radiation dose was relatively low (mean dose: 1,326 cGy), and the RT course was discontinued before completion, making RT a less likely primary driver of the acute, severe myocardial injury. However, a potential interaction between RT and ICIs cannot be entirely excluded. RT is known to induce immunogenic cell death and to augment both local and systemic immune responses, which may synergize with ICIs and lower the threshold for severe irAEs in susceptible individuals (14, 15). Future research should prioritize the identification of predictive biomarkers for severe irAEs in high-risk populations, particularly those with pre-existing autoimmune diseases such as MG. In parallel, the development of multidisciplinary management pathways to facilitate early detection and aggressive treatment of these life-threatening complications is essential.

In this case, the patient underwent comprehensive multidisciplinary evaluation and management. From a cardiology perspective, the extent of myocardial injury was considered substantial, involving both ventricles and extending from the endocardium to the epicardium, with an overall poor prognosis. Cardiac magnetic resonance imaging or endomyocardial biopsy would have provided a more definitive diagnosis; however, cardiac MRI could not be completed due to ventricular arrhythmias (frequent premature ventricular contractions), and endomyocardial biopsy was not performed because of its invasiveness and the patient’s unstable condition. Based on the temporal relationship with PD-1 inhibitor therapy, the marked elevation of cardiac biomarkers, and the characteristic ECG and echocardiographic changes, the team concluded that the patient had extensive ICI-associated myocardial injury. Although ICI-related myocarditis is not exceedingly rare, fulminant presentations such as in this case are uncommon. The cardiology team also considered the current presentation to be unrelated to the patient’s prior history of ischemic heart disease.

Radiation oncology specialists assessed that the patient had locally advanced esophageal cancer and had been treated with chemoradiotherapy according to contemporary guidelines. Because the tumor was a medullary-type carcinoma—typically less radiosensitive—and extended over an 8 cm segment of the esophagus, the expected efficacy of chemoradiotherapy alone was limited, and PD-1 inhibitor therapy was added to enhance tumor control. Given the relatively low mean cardiac radiation dose and incomplete RT course, together with the typical time window for ICI myocarditis (commonly 17–65 days after the first PD-1 dose and independent of cumulative drug dose), the multidisciplinary team considered immunotherapy-related myocardial injury to be the most likely cause of the acute cardiac deterioration. In contrast, RT-induced myocardial damage is usually associated with higher cumulative doses and longer latency; therefore, the patient’s current condition was considered unlikely to be attributable to RT.

The oncology team reviewed the patient’s prior anticancer treatment and its temporal relationship to PD-1–induced myocardial toxicity, and recommended initiation of high-dose corticosteroids to attenuate the immune-mediated myocardial inflammation (methylprednisolone 500–1,000 mg once daily for 3–5 days). At the same time, acute liver and kidney injury were recognized, and artificial liver support and dialysis were instituted. Synthesizing the opinions of cardiology, oncology, radiation oncology, and critical care, the patient was diagnosed with severe ICI-associated myocarditis accompanied by hemodynamic instability and multiorgan dysfunction of varying severity. High-dose methylprednisolone (500 mg once daily for 3–5 days) was administered and subsequently tapered under close clinical and laboratory monitoring. According to the final multidisciplinary consensus, the patient’s presentation was consistent with ICI-related myocarditis on a background of MG. Serial laboratory tests showed an initial improvement in cardiac biomarkers; however, the overall condition remained critical, with progressive multiorgan failure. Despite aggressive treatment, organ function did not recover, and the patient ultimately suffered cardiac arrest and died.

For high-risk populations such as patients with MG undergoing combined RT and immunotherapy, rigorous monitoring of cardiac status is essential. Key parameters include troponin, NT-proBNP, left ventricular ejection fraction (LVEF), CK and CK-MB, as well as serial ECGs. Troponin is a highly specific marker of myocardial injury, and elevated levels are usually directly related to damage to cardiomyocytes. Studies have demonstrated that hs-TnT offers greater sensitivity for detecting myocarditis compared with other cardiac biomarkers such as CK and cTnI, making it a priority in routine surveillance. Furthermore, dynamic changes in hs-TnT correlate closely with the severity of myocardial injury and clinical outcomes, aiding clinicians in assessing disease progression and treatment response. In the context of ICI-related myocarditis, elevations in NT-proBNP are often associated with structural and functional impairment of the heart and provide additional support for the diagnosis. Echocardiography enables quantitative assessment of left ventricular systolic and diastolic function, thereby helping to grade the severity of myocarditis and evaluate overall cardiac performance; a reduction in LVEF typically correlates with more advanced myocardial damage (16, 17).

When managing cardiotoxicity in patients with MG, individual patient factors—including baseline functional status, comorbidities, and treatment tolerance—must be carefully considered. This may necessitate tailoring the RT dose, modifying or temporarily suspending immunotherapy, or introducing cardioprotective strategies. In such high-risk populations, optimal care requires close multidisciplinary collaboration among radiation oncologists, medical oncologists, cardiologists, and neurologists. Such collaboration is crucial for early recognition, timely diagnosis, and aggressive management of myocarditis and related irAEs. Equally important is the proactive identification of high-risk patients to facilitate prevention, risk stratification, and more intensive monitoring for ICI-related myocarditis.

## Conclusion

This case describes a 69-year-old man with esophageal squamous cell carcinoma and pre-existing MG who developed ICI–associated myocarditis during combined RT and immunotherapy. Despite prompt initiation of high-dose corticosteroids and intensive multidisciplinary management, the patient ultimately succumbed to multiorgan failure. The diagnosis and treatment of myocarditis were complicated by the interplay of pre-existing MG, prior cardiac disease, and concurrent oncologic therapies. This case underscores the need for meticulous cardiac surveillance in patients with autoimmune comorbidities receiving ICI-based regimens, and highlights the critical role of multidisciplinary collaboration in the early detection and management of severe immune-related adverse events.

## Data Availability

The original contributions presented in the study are included in the article/supplementary material. Further inquiries can be directed to the corresponding authors.

## References

[1] Lipe DN Qdaisat A Krishnamani PP Nguyen TD Chaftari P El Messiri N . Myocarditis, myositis, and myasthenia gravis overlap syndrome associated with immune checkpoint inhibitors: a systematic review. Diagn (Basel). (2024) 14:1794. doi: 10.3390/diagnostics14161794, PMID: 39202282 PMC11353298

[2] Wang F Yang S Palmer N Fox K Kohane IS Liao KP . Real-world data analyses unveiled the immune-related adverse effects of immune checkpoint inhibitors across cancer types. NPJ Precis Oncol. (2021) 5:82. doi: 10.1038/s41698-021-00223-x, PMID: 34508179 PMC8433190

[3] Pathak R Katel A Massarelli E Villaflor VM Sun V Salgia R . Immune checkpoint inhibitor-induced myocarditis with myositis/myasthenia gravis overlap syndrome: a systematic review of cases. Oncologist. (2021) 26:1052–61. doi: 10.1002/onco.13931, PMID: 34378270 PMC8649039

[4] Anscher MS Arora S Weinstock C Amatya A Bandaru P Tang C . Association of radiation therapy with risk of adverse events in patients receiving immunotherapy: a pooled analysis of trials in the US Food and Drug Administration database. JAMA Oncol. (2022) 8:232–40. doi: 10.1001/jamaoncol.2021.6439, PMID: 34989781 PMC8739815

[5] Cheng W Sun T Liu C Zhou Z Duan J Zhao Y . A systematic review of myasthenia gravis complicated with myocarditis. Brain Behav. (2021) 11:e2242. doi: 10.1002/brb3.2242, PMID: 34105901 PMC8413805

[6] Zhang C Bu B Yang H Wang L Liu W Duan R-S . Immunotherapy choice and maintenance for generalized myasthenia gravis in China. CNS Neurosci Ther. (2020) 26:1241–54. doi: 10.1111/cns.13468, PMID: 33103369 PMC7702233

[7] Salem J-E Manouchehri A Moey M Lebrun-Vignes B Bastarache L Pariente A . Cardiovascular toxicities associated with immune checkpoint inhibitors: an observational, retrospective, pharmacovigilance study. Lancet Oncol. (2018) 19:1579–89. doi: 10.1016/S1470-2045(18)30608-9, PMID: 30442497 PMC6287923

[8] Moreira A Loquai C Pföhler C Kähler KC Knauss S Heppt MV . Myositis and neuromuscular side-effects induced by immune checkpoint inhibitors. JAMA Oncol. (2019) 5:906–11. doi: 10.1001/jamaoncol.2019.0362, PMID: 30453170

[9] Lipe DN Galvis-Carvajal E Rajha E Wechsler AH Gaeta S . Immune checkpoint inhibitor-associated myasthenia gravis, myositis, and myocarditis overlap syndrome. Am J Emerg Med. (2021) 46:51–5. doi: 10.1016/j.ajem.2021.03.005, PMID: 33721590

[10] Matas-García A Martinez-Hernandez E Milisenda JC . Treatment of myositis associated with immune checkpoint inhibitors. Curr Treat Options Rheumatol. (2023) 9:179–91. doi: 10.1007/s40674-023-00212-0

[11] Seuthe K Pfister R Pennig L Mons U Klingel K Ten Freyhaus H . Endomyocardial biopsy in patients with myocarditis-still justified in the CMR era? A single-centre experience. Clin Res Cardiol. (2024). doi: 10.1007/s00392-024-02574-4, PMID: 39570399 PMC13083329

[12] Gu J Qiao Y Huang R Cong S . Efficacy and safety of immunosuppressants and monoclonal antibodies in adults with myasthenia gravis: a systematic review and network meta-analysis. J Transl Med. (2024) 22:955. doi: 10.1186/s12967-024-05751-1, PMID: 39434135 PMC11492773

[13] Sobrino TM Dolladille C González-Jiménez P Dompmartin A Ezine E Sassier M . Myasthenia gravis and adverse events from immune checkpoint inhibitors: an international pharmacovigilance study. Eur Heart J. (2024) 42(48):4964–77. doi: 10.1093/eurheartj/ehaf315, PMID: 34529770

[14] Bardoscia L Pasinetti N Triggiani L Cozzi S Sardaro A . Biological bases of immune-related adverse events and potential crosslinks with immunogenic effects of radiation. Front Pharmacol. (2021) 12:746853. doi: 10.3389/fphar.2021.746853, PMID: 34790123 PMC8591245

[15] Tan S Li D Zhu X . Cancer immunotherapy: Pros, cons and beyond. BioMed Pharmacother. (2020) 124:109821. doi: 10.1016/j.biopha.2020.109821, PMID: 31962285

[16] Power JR Dolladille C Ozbay B Procureur AM Ederhy S Palaskas NL . Predictors and risk score for immune checkpoint-inhibitor-associated myocarditis severity. medRxiv. (2024). doi: 10.1101/2024.06.02.24308336, PMID: 38883792 PMC11177901

[17] Lu X Peng Q Wang G . Discovery of new biomarkers of idiopathic inflammatory myopathy. Clin Chim Acta. (2015) 444:117–25. doi: 10.1016/j.cca.2015.02.007, PMID: 25681646

